# Is soft drink consumption associated with gestational hypertension? Results from the BRISA cohort

**DOI:** 10.1590/1414-431X202010162

**Published:** 2021-01-22

**Authors:** J.M.A. Barbosa, A.A.M. da Silva, G. Kac, V.M.F. Simões, H. Bettiol, R.C. Cavalli, M.A. Barbieri, C.C.C. Ribeiro

**Affiliations:** 1 Departamento de Saúde Pública, Universidade Federal do Maranhão, São Luís, MA, Brasil; 2 Departamento de Nutrição Social Aplicada, Instituto de Nutrição Josué de Castro, Universidade Federal do Rio de Janeiro, Rio de Janeiro, RJ, Brasil; 3 Departamento de Puericultura e Pediatria, Faculdade de Medicina de Ribeirão, Universidade de São Paulo, Ribeirão Preto, SP, Brasil; 4 Departamento de Ginecologia e Obstetrícia, Faculdade de Medicina de Ribeirão Preto, Universidade de São Paulo, Ribeirão Preto, SP, Brasil; 5 Departamento de Odontologia II, Universidade Federal do Maranhão, São Luís, MA, Brasil

**Keywords:** Gestational hypertensive disorders, Soft drink consumption, Cohort study, Marginal structural model, Pregnancy

## Abstract

It is still unknown whether excessive consumption of sugar-sweetened beverages may be linked to gestational hypertensive disorders, other than preeclampsia. This study investigated the association between soft drink consumption and hypertension during pregnancy, analyzing the relationship from the perspective of counterfactual causal theory. Data from pregnant women of the BRISA cohort were analyzed (1,380 in São Luis and 1,370 in Ribeirão Preto, Brazil). The explanatory variable was the frequency of soft drink consumption during pregnancy obtained in a prenatal interview. The outcome was gestational hypertension based on medical diagnosis, at the time of delivery. A theoretical model of the association between soft drink consumption and gestational hypertension was constructed using a directed acyclic graph. Marginal structural models (MSM) weighted by the inverse of the probability of soft drink consumption were also employed. Using Poisson regression analysis, high soft drink consumption (≥7 times/week) was associated with gestational hypertension in São Luís (RR=1.48; 95%CI: 1.03-2.10), in Ribeirão Preto (RR=1.51; 95%CI: 1.13-2.01), and in the two cohorts combined (RR=1.45; 95%CI: 1.16-1.82) compared to lower exposure (<7 times/week). In the MSM, the association between high soft drink consumption and gestational hypertension was observed in Ribeirão Preto (RR=1.63; 95%CI: 1.21-2.19) and in the two cohorts combined (RR=1.51; 95%CI: 1.15-1.97), but not in São Luís (RR=1.26; 95%CI: 0.79-2.00). High soft drink consumption seems to be a risk factor for gestational hypertension, suggesting that it should be discouraged during pregnancy.

## Introduction

Hypertensive disorders of pregnancy (HDP) include: 1) chronic hypertension (of any cause that precedes pregnancy); 2) gestational hypertension (high blood pressure (BP) after 20 weeks of gestation in the absence of preeclampsia); 3) preeclampsia-eclampsia (high BP after 20 weeks of gestation with proteinuria or any of the severe features of preeclampsia); and 4) chronic hypertension with superimposed preeclampsia (chronic hypertension in association with preeclampsia) (1). HDP are a major cause of maternal mortality and perinatal outcomes such as prematurity, perinatal asphyxia, and low birth weight (1), and also increase the risk of future diseases in the mother and child (2).

Dietary factors seem to play an important role in the etiology of HDP (3,4). High scores of the Healthy Eating Index adapted for use in pregnant women have been shown to protect against preeclampsia (OR=0.87; 95%CI: 0.76-1.00) (3). A meta-analysis concluded that women with high energy intake were at higher risk of developing preeclampsia compared to their counterparts (4). The main source of energy intake during pregnancy is excessive sugar consumption, especially soft drinks (5). High soft drink consumption has been associated with overweight/obesity, metabolic syndrome, and type II diabetes (6). All these metabolic disorders are associated to HDP (7). Systematic reviews (8,9) have shown that high consumption of soft drinks and other sugar-sweetened beverages (SSBs) are associated with increased BP and hypertension in non-pregnant women.

Gestational hypertension has a faster evolution and distinct pathophysiological mechanisms compared to hypertension outside pregnancy. Metabolic adaptations are present in pregnancy and inflammatory responses are exacerbated in gestational hypertension (10). Therefore, it is biologically plausible that the risk of high soft drink consumption associated with gestational hypertension is somewhat different from the risk of high soft drink consumption associated with hypertension outside pregnancy.

To the best of our knowledge, only two studies conducted on pregnant women have found that high daily sugar intake in pregnancy was associated with preeclampsia (5,11). Furthermore, when considering the association between added sugars or SSBs and preeclampsia separately, the association was strongest for SSBs (5). Another study pointed out that intake of added sugars is a risk factor for preeclampsia, but this association lost statistical significance in the multiple logistic regression model, and only the association between SSBs and preeclampsia remained significant (OR=1.33; 95%CI: 1.10- 1.61) (11).

High intake of soft drinks during pregnancy, analyzed by conventional regression models, seems to be associated with more severe hypertension (preeclampsia) (5,11). The present study was conducted to investigate the association between soft drink consumption during pregnancy and gestational hypertension in a prenatal cohort, analyzing these relationships from the perspective of counterfactual causal theory.

## Material and Methods

This study is part of a large research project that aimed to evaluate risk factors for preterm birth in a cohort of pregnant women selected during the first weeks of pregnancy named BRISA (12) (Brazilian Ribeirão Preto and São Luís Birth Cohort Studies, Portuguese acronym). This cohort was carried out in two cities: São Luís, in the Brazilian Northeast and Ribeirão Preto, in the Brazilian Southeast.

A convenience sample was carried out due to the lack of registries of pregnant women or women receiving prenatal care that were necessary to obtain a random sample representing the pregnant women population in São Luís and in Ribeirão Preto; thus, the reference population consisted of pregnant women users of prenatal care of all major public and private hospitals in both cities. At baseline, the pregnant women were invited for an interview that was held from the 22nd to the 25th week of gestation. At delivery (follow-up), the women were reinterviewed after the birth of their babies.

Inclusion criteria were having been submitted to obstetric ultrasound before the 20th week of pregnancy in order to determine gestational age (GA) (13) and singleton pregnancy.

### Data collection

Data were collected during an interview when a structured questionnaire was applied and clinical examinations in the prenatal period (baseline) and at delivery (from January 2010 to June 2011) were performed. During the prenatal period, 1,447 women were interviewed in the São Luís cohort and 1,400 in the Ribeirão Preto cohort. A total of 1,381 women in São Luís and 1,370 in Ribeirão Preto were interviewed again during the first 24 h after delivery. One pregnant woman in São Luís was excluded from the study because she did not provide information about hypertension during pregnancy.

The following information was collected in the prenatal period: age, family income, smoking habit during pregnancy, daily and weekly soft drink consumption, reported weight before pregnancy, and height measured with an Altura Exata¯ (TBW, Brazil) stadiometer. Additional information was obtained after delivery: schooling, skin color, parity, and hypertension based on medical diagnosis.

### Exposure variables, confounding factors, mediators, and outcome

The exposure was soft drink consumption during pregnancy up to 22/25 weeks of GA. This information was obtained from answers to the following two questions: 1) On how many days of the week do you consume soft drinks? 2) How many times a day do you take soft drinks? The frequencies of weekly intake (zero to seven days a week) and of daily intake (once to six times a day) of soft drinks reported by the women were multiplied and categorized as a dichotomous variable corresponding to no or low consumption (<7 times per week) or high consumption (≥7 times per week) (14). This cutoff point was assumed since the majority of previous studies has shown that daily SSBs intake is associated to worse health outcomes, such as obesity and hypertension (8,9,15). Tertiles of soft drink consumption (measured in times per week) were also calculated to test a dose-response gradient in the association between soft drink consumption and gestational hypertension, as follows: for São Luís: 1st tertile (no consumption), 2nd tertile (once a week), and 3rd tertile (two or more times per week); and for Ribeirão Preto: 1st tertile (up to once a week), 2nd tertile (two to six times per week), and 3rd tertile (seven or more times per week).

The outcome was hypertension during the current pregnancy based on medical diagnosis (yes or no). This information was obtained from the response to the following question: “Did you have hypertension (high blood pressure) in the current pregnancy, diagnosed by a doctor or a nurse?”

Sociodemographic and economic variables were considered to be confounding factors since these variables are ancestral (common factors) regarding both exposure (16) and outcome (3). Maternal age was categorized as 14-19, 20-24, 25-29, 30-34, and ≥35 years. Skin color was self-reported and categorized as white, black, brown (which represents miscegenation of black and white Brazilians), and yellow (Asian peoples).

Maternal schooling was categorized as ≤4, 5-8, 9-11, and ≥12 years. Family income was divided into tertiles as follows: for São Luís: 1st tertile (US$ 446.80), 2nd tertile (US$ 755.80), and 3rd tertile (US$ 1,162.70); and for Ribeirão Preto: 1st tertile (US$ 552.30), 2nd tertile (US$ 872.10), and 3rd tertile (US$ 1,279.10), considering a US dollar exchange value of 1.7 for the Brazilian real (July 2010). Parity was categorized as 1, 2-3, and ≥4 deliveries. Smoking habit in the current pregnancy was categorized as yes or no. Pre-gestational body mass index (BMI) was calculated according to the WHO (World Health Organization) criterion (17) and assigned to three categories: ≤24.9, 25-29.9, or ≥30 kg/m^2^.

Junk food intake was considered a potential confounding factor for the association between soft drink consumption and gestational hypertension since these foods are usually consumed together with soft drinks and are associated with higher BMI (18). Junk food intake was based on consumption of the following items: hamburger, cheeseburger, or cheese and meat sandwich, sausage, hot dog and salami, ham, baloney, potato chips, packaged salty snacks, and popcorn. The frequency of consumption of each junk food item was first categorized as follows: up to three times a month, once to twice a week, and ≥3 times a week. The sum of each of these categories of consumption was calculated for all food items and was then divided into tertiles as follows: for both São Luís and Ribeirão Preto, 1st tertile (up to three times a month), 2nd tertile (once to twice a week), and 3rd tertile (≥3 times a week).

### Theoretical model and data analysis

Two analytical strategies were employed to answer the question of the present study. Causal diagrams were first elaborated to guide the analysis of the effect of soft drink consumption on hypertension during pregnancy, with control for confounding factors in the most appropriate manner and at the same time avoiding unnecessary adjustments (19). Second, marginal structural models (MSM) estimated with weighting by the inverse of the probability of soft drink consumption were used to estimate the association between soft drink consumption and hypertension during pregnancy. MSM are used for the causal analysis of observational data as long as the assumptions of exchangeability, positivity, well-defined exposures, and a correct model specification are satisfied (20). A theoretical model was constructed to analyze the association between soft drink consumption and gestational hypertension using directed acyclic graphs (DAG) elaborated using Dagitty 2.2¯ (<http://www.dagitty.net>) software.

**Figure 1:**
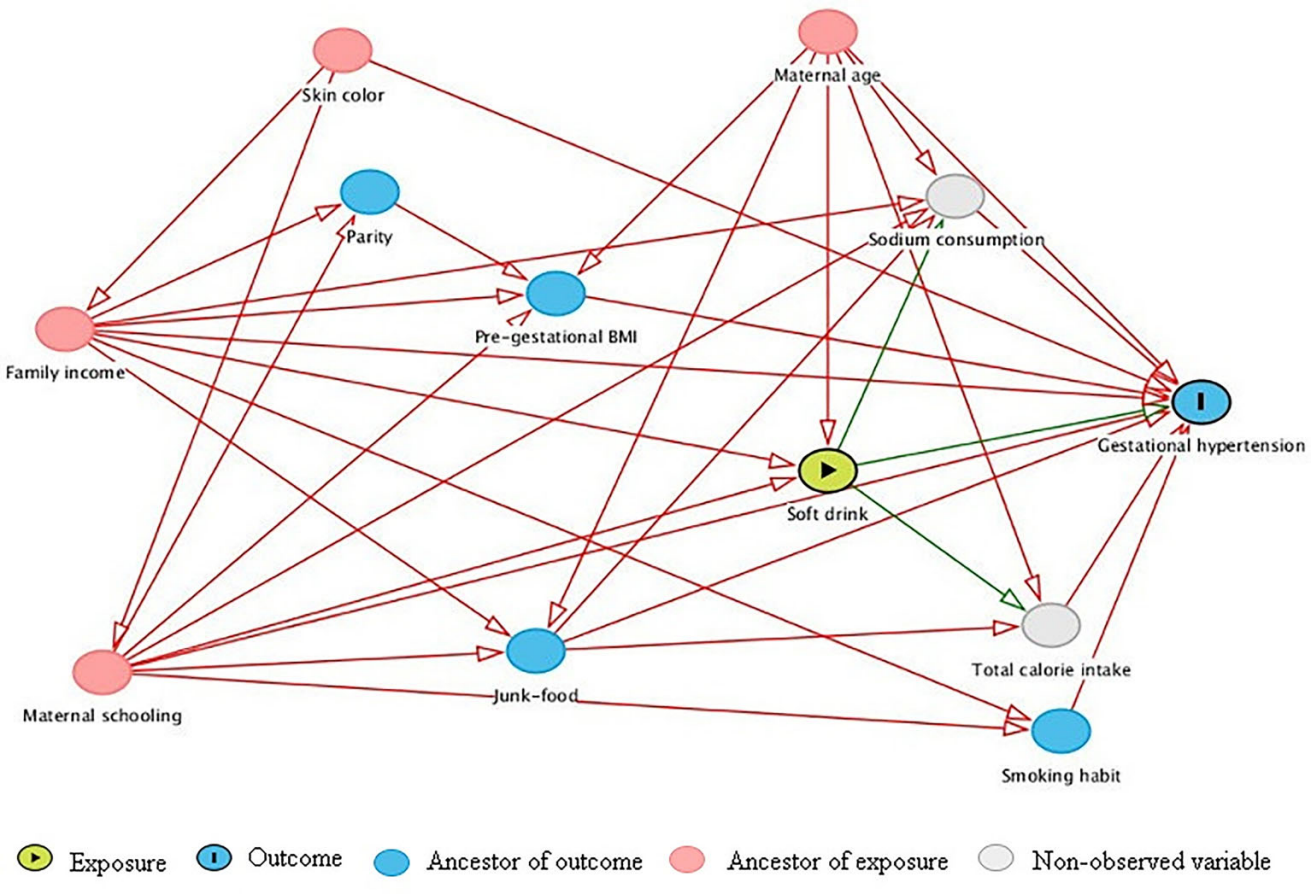
Theoretical model of the association between soft drink consumption and hypertension during the current pregnancy based on a directed acyclic graph (DAG).

In addition to the relationships between the observed variables described above, two non-observed variables, salt consumption and total energy intake, were included in the theoretical model of association between soft drink consumption and gestational hypertension, since they might be important confounders (1,4). Since soft drinks are important sources of sodium and of discretionary calories (6), these two variables were considered as mediators of the effect of soft drinks on gestational hypertension (Figure 1).

To estimate the total effect (non-mediated effect) of the association between soft drink consumption and hypertension during pregnancy, the following variables were suggested to be included in the minimally sufficient adjustment set for confounding, by the application of the back-door criterion: maternal schooling, family income, and age of the pregnant women. According to the back-door criterion, to estimate this effect, skin color, parity, BMI, junk food consumption, and smoking should not enter the model.

The chi-squared test was performed to compare the characteristics between pregnant women in the São Luís and Ribeirão Preto cohorts, considering a P-value <0.05 as statistically significant. Poisson regression analysis with robust adjustment of variance was used to test the association between soft drink consumption and hypertension during the current pregnancy. The level of significance was set at 0.05. Risk ratios and their respective confidence intervals (95%CI) were estimated.

For the MSM analysis, we first estimated a logistic regression model to predict soft drink consumption including as predictors variables significantly associated with this exposure (age, schooling, junk food consumption, and smoking habit during pregnancy) (Table 1). Based on this model, we calculated the probability of soft drink consumption for each individual. A marginal structural model (Poisson regression with robust standard errors) based on the counterfactual logic including soft drink consumption during pregnancy as the exposure and gestational hypertension as the outcome was then estimated, weighted by the inverse of the probability of soft drink consumption. All analyses were carried out using STATA software version 12.0 (StataCorp, USA).

**Table 1:** Descriptive characteristics of the pregnant women, comparing the São Luís and Ribeirão Preto cohorts, 2010-2011.

Gestational hypertension	São Luís (n, %)	Ribeirão Preto (n, %)	Chi-squared P-value
Age (years)			<0.001
14–19	169 (12.3)	215 (15.7)	
20–24	449 (32.5)	387 (28.3)	
25–29	424 (30.7)	417 (30.4)	
30–34	243 (17.6)	221 (16.1)	
≥35	95 (6.9)	130 (9.5)	
Schooling (years)			0.070
≤4	24 (1.7)	57 (4.2)	
5–8	146 (10.6)	337 (24.6)	
9–11	1056 (76.5)	862 (62.9)	
≥12	154 (11.2)	114 (8.3)	
Family income (tertiles)			0.326
1st	450 (33.6)	413 (33.4)	
2nd	445 (33.2)	413 (33.4)	
3rd	445 (33.2)	412 (33.2)	
Skin color			0.077
White	223 (16.2)	700 (51.5)	
Black	208 (15.1)	152 (11.2)	
Brown	925 (68.0)	500 (36.8)	
Yellow	23 (1.67)	6 (0.5)	
Parity (number of deliveries)			0.133
1	711 (51.5)	676 (49.3)	
2–3	592 (42.9)	593 (43.3)	
≥4	77 (5.6)	101 (7.4)	
Smoking habit			0.100
No	1321 (95.7)	1190 (87.1)	
Yes	59 (4.3)	176 (12.9)	
Pre-gestational BMI^a^			<0.001
Normal weight	941 (74.5)	809 (61.2)	
Overweight	248 (19.6)	329 (24.9)	
Obesity	75 (5.9)	185 (13.9)	
Soft drink consumption^b^			0.039
<7 times per week	1243 (90.9)	943 (69.6)	
≥7 times per week	124 (9.1)	411 (30.4)	
Junk food consumption (tertiles)^c^			0.222
1st	654 (47.5)	519 (37.9)	
2nd	419 (30.4)	463 (33.9)	
3rd	306 (22.1)	385 (28.2)	
Gestational hypertension			<0.001
No	1147 (83.1)	1178 (86.0	
Yes	233 (16.9)	192 (14.0)	

Numbers may not add up to total because of missing values. ^a^Body mass index: normal weight: ≤24.9 kg/m^2^, overweight 25–29.9 kg/m^2^, and obesity ≥30 kg/m^2^. ^b^Multiplying times per day *vs* days per week and reported in times per week. ^c^Tertile of junk food consumption classification for both São Luís and Ribeirão Preto: 1st tertile (up to three times a month), 2nd tertile (once to twice a week), and 3rd tertile (≥3 times a week).

### Ethical aspects

The study was approved by the Research Ethics Committee of the University Hospital of the Federal University of Maranhão (#4771/2008-30) and by the Research Ethics Committee of the University Hospital, Faculty of Medicine of Ribeirão Preto, University of São Paulo (#4116/2008).

## Results

In São Luís, 16.9% of the 1,380 pregnant women reported gestational hypertension and 9.1% consumed soft drinks ≥7 times per week. In the Ribeirão Preto cohort, 14.0% of the 1,370 pregnant women were hypertensive and 30.4% consumed soft drinks ≥7 times per week (Table 1). The median value of consumption in Ribeirão Preto was 6 times per week, while in São Luís the median value of consumption was 2 times per week (data not shown in tables).

Age, schooling, junk food consumption, and smoking habit were predictors of soft drink consumption by the pregnant women (Supplementary Table S1).

In the Poisson regression analysis, soft drink consumption ≥7 times per week was associated with gestational hypertension in São Luís (RR=1.48; 95%CI: 1.03-2.10; P=0.030), in Ribeirão Preto (RR=1.51; 95%CI: 1.13-2.01; P=0.005), and in the two cohorts combined (RR=1.45; 95%CI: 1.16-1.82; P=0.001) (Supplementary Table S2).

In the MSM, soft drink consumption ≥7 times per week was significantly associated with hypertension in the Ribeirão Preto cohort (RR=1.63; 95%CI: 1.21-2.19; P=0.001) and in the two cohorts combined (RR=1.51; 95%CI: 1.15-1.97; P=0.002), but not in the São Luís cohort alone (RR=1.26; 95%CI: 0.79-2.00; P=0.328) (Table 2).

**Table 2:** Marginal structural models to estimate the association of soft drink consumption with hypertension during pregnancy in the São Luís and Ribeirão Preto cohorts, 2010-2011.

Gestational	São Luís			Ribeirão Preto			São Luís and Ribeirão Preto		
hypertension	RR	95%CI	P-value	RR	95%CI	P-value	RR	95%CI	P-value
≥7 times/week	1.26	0.79–2.00	0.328	1.63	1.21–2.19	0.001	1.51	1.15–1.97	0.039
2nd tertile	1.08	0.79–1.48	0.589	0.91	0.64–1.30	0.622	1.19	0.92–1.54	0.182
3rd tertile	1.35	1.02–1.78	0.032	1.45	1.04–2.01	0.026	1.26	1.01–1.57	0.038
Dose response	1.16	1.01–1.33	0.036	1.20	1.00–1.44	0.039	1.12	1.00–1.25	0.033

RR: risk ratio; 95%CI: confidence interval. Only variables with a P-value <0.05 were considered to be statistically significant.

Soft drink consumption divided into tertiles was also tested to verify the consistency of our findings. The lowest tertile of exposure was compared with the two other categories (medium and highest tertiles). The highest tertile of soft drink consumption was associated with gestational hypertension in São Luís (RR=1.35; 95%CI: 1.02-1.78; P=0.032), in Ribeirão Preto (RR=1.45; 95%CI: 1.04-2.01; P=0.026), and in the two cohorts combined (RR=1.26; 95%CI: 1.01-1.57; P=0.038). A dose-response relationship was also demonstrated between soft drink consumption and gestational hypertension in São Luís (RR=1.16; 95%CI: 1.01-1.33; P=0.036), in Ribeirão Preto (RR=1.20; 95%CI: 1.00-1.44; P=0.039), and in the two cohorts combined (RR=1.12; 95%CI: 1.00-1.25; P=0.033) (Table 2).

## Discussion

The results of the present study suggested that high soft drink consumption (≥7 times per week) influenced gestational hypertension, even after adjustment for confounding variables chosen based on the back-door criterion in a DAG. High soft drink consumption was associated with gestational hypertension in each city of the BRISA cohort and also in the combined analysis of the two cities. A dose response gradient between soft drink consumption and gestational hypertension was also observed.

An association between high soft drink consumption and gestational hypertension for the Ribeirão Preto cohort and in the joint analysis of the two cities was also present in the MSM, suggesting a causal effect. However, for the São Luís cohort no association between high soft drink consumption and gestational hypertension was detected in the MSM. It is important to note that the group exposed to high soft drink consumption in São Luís (9.1%) was smaller compared to Ribeirão Preto (30.4%) and this may help explain this difference between the two cities. In addition, the median value of the consumption in Ribeirão Preto was 3 times higher than the median value in São Luís, a fact that may also help explain the present findings.

Association of high soft drink consumption and hypertension has been reported in non-pregnant women (21). Our results suggested that soft drink consumption also seemed to be a risk factor for HDP, confirming what has been shown in systematic review of studies including non-pregnant women (8,9).

Hormonal and metabolic abnormalities occur naturally during gestation resulting in increased low-density lipoprotein (LDL)-cholesterol and triglyceride levels, and also in decreased high-density lipoprotein (HDL)-cholesterol (22). These physiological adaptations increase oxidative stress during pregnancy that may result in a higher risk of hypertension (23). Oxidative stress in gestation may be unbalanced by excessive pregestational weight and by an unhealthy diet (high sugar/high fatty content) resulting in oxidative damage (24). Furthermore, SSBs have been consistently implicated in increased triglycerides and HDL-cholesterol levels in randomized clinical trials (25). Furthermore, those beverages have been clearly associated to obesity risk (15).

A possible effect of high soft drink consumption on gestational hypertension was suggested in the MSM. Previous studies have already suggested that soft drink consumption may be associated with more severe signs and symptoms of HDP. However, these results were obtained by studying associations between total energy consumption (1) or sugar consumption (10) and preeclampsia using conventional regression models.

Interestingly, cohort studies have also associated high SSB consumption during pregnancy with preterm birth (26). Since maternal hypertension is a recognized risk factor for preterm birth (27), these data may suggest that soft drink consumption is a common risk factor for both HDP and preterm birth.

A possible mechanism to explain the association shown here between high soft drink consumption and gestational hypertension is the high sucrose content of soft drinks, which may contain added fructose from invert sugars (28). Greater consumption of added fructose has been associated with increased inflammatory response, dyslipidemia, insulin resistance, and increased systolic BP (29). Randomized clinical trials have shown that SSB consumption for three weeks results in increased C-reactive protein and is associated with metabolic disorders (dyslipidemia and insulin resistance) (30). Furthermore, intake of sugary drinks is associated with increased mean BP (31). These inflammatory and metabolic disorders that are linked to obesity, dyslipidemia, and insulin resistance are also present in HDP (32). Studies have shown little difference in the rate of increased weight gain between consumption of high fructose corn syrup and sucrose derivatives from sugarcane (33); furthermore being overweight can increase oxidative stress during pregnancy (24) as well as gestational hypertension (34).

In the physiopathology of HDP there is an angiogenic imbalance, with involvement of oxidative stress and endothelial dysfunction (32). The mechanism of the association between high soft drink consumption and gestational hypertension shown here might be mediated by vascular changes. Supporting this hypothesis, consumption of added fructose has been shown to be associated with endothelial dysfunction, reduction of protective vascular factors, and increased oxidative stress, metabolic disorders, and high BP in an animal model (35).

Our data did not distinguish whether soft drinks were sugar- or artificially-sweetened. However, it is much more probable that consumption of the caloric version predominated, since in Brazil only 11% of the population consume exclusively the artificially sweetened version (36). Artificially sweetened drinks have also been implicated in systemic metabolic changes, such as excessive weight gain, metabolic syndrome (26), hypertension (37), and preterm birth (26) through still unknown mechanisms. Thus, we cannot totally discard that other mechanisms not triggered by added sugars could be involved in the association between soft drinks consumption and hypertension shown here. The high sodium content of soft drinks (6) may also be a mechanism capable of explaining our results, considering that sodium retention is also characteristic of HDP (38). However, the effect of restricted salt consumption on HDP has not been supported by evidence (1).

It is possible that the association detected here between high soft drink consumption and gestational hypertension may be spurious, with soft drink consumption only representing an inappropriate dietary pattern. However, adjustment for junk food consumption in the MSM minimized the possibility that this association between greater soft drink consumption and gestational hypertension would only reflect other inappropriate eating habits.

A limitation of the current study is that no adjustment was made for dietary data that may also be involved in the association between soft drink consumption and gestational hypertension, especially sodium consumption and total calorie intake, information that was not available in the studies. However, these non-observed variables were included in the theoretical model and, according to the back-door criterion, adjustment for these variables to estimate the total effect of soft drink consumption on gestational hypertension would not be necessary. Another limitation is the impossibility of distinguishing between different HDP types. Thus, it was not possible to exclude the possibility that preeclampsia was the only disorder associated with high soft drink consumption. Furthermore, aggregation of data for some categories of maternal schooling might have decreased our ability to find differences between categories of maternal schooling according to soft drink consumption and might thus have decreased our ability to adjust for confounding.

Total intake of soft drinks during pregnancy would also be relevant but it has not been assessed. However, daily frequency of SSBs consumption has been the exposure most frequently studied in the meta-analyses that showed association between SSB and hypertension in non-pregnant populations (8,9). As a limitation, soft drink consumption was assessed only once at 22/25 weeks of gestation; thus, we cannot be sure that the same frequency of this unhealthy behavior had been maintained throughout the remaining gestational period. Exposure to high soft drink intake in the beginning of gestation was consistently associated with gestational hypertension in the two birth cohorts, showing a dose-response gradient. Finally, the non-probabilistic convenience sample of our study was prone to selection bias, limiting the external generalizability of the findings; however, our results showed consistency in the association between soft drink consumption during gestation and gestational hypertension in two different Brazilian cities.

Reporting of hypertension based on medical diagnosis may also be pointed out as a potential source of information bias. However, this information was considered to be reliable since all pregnant women of the two cohorts attended more than six prenatal visits. In addition, prevalence rates of gestational hypertension reported in the two cities were similar to those obtained in public maternity hospitals in Brazil (39) and in one cohort study conducted in the United Kingdom (40).

An important strength of the present study was its prospective design, in which soft drink consumption was measured before the outcome. Another positive aspect was the construction of a theoretical causal model based on the back-door criterion in a DAG, which allowed us to select an appropriate minimum set of confounders. The use of the MSM increased our ability to make causal inference regarding the association detected here between soft drink consumption and gestational hypertension, as long as the assumptions for causal interpretation had been fulfilled.

Greater soft drink consumption seemed to be a risk factor for gestational hypertension regardless of adjustment for socioeconomic factors, smoking, and junk food consumption, suggesting that high consumption of these beverages should be discouraged during pregnancy. Obstetric advice should include guidance on healthy eating habits for pregnant women emphasizing the need to restrict consumption of these beverages rich in added sugars in order to avoid metabolic disorders and the development of gestational hypertension.

## Supplementary Material

Click here to view [pdf].
